# Early B lymphocyte subsets in blood predict prognosis in sepsis

**DOI:** 10.3389/fimmu.2024.1437864

**Published:** 2024-09-18

**Authors:** Yingqian Sun, Yan Lu, Xinling Pan, Chengliang Zhang, Liang Wang, Longyi Zhang

**Affiliations:** ^1^ Clinical Laboratory, Affiliated Dongyang Hospital of Wenzhou Medical University, Dongyang, Zhejiang, China; ^2^ Department of Biomedical Sciences Laboratory, Affiliated Dongyang Hospital of Wenzhou Medical University, Dongyang, Zhejiang, China

**Keywords:** sepsis, B lymphocytes, B cell subset, flow cytometry, prognosis, CD5^+^ B cells, innate immune, double-negative B cells

## Abstract

**Background:**

B lymphocytes play a key role in immunosuppression. This study investigated the prognostic value of B cell subsets in sepsis.

**Methods:**

Flow cytometry was used to assess peripheral B cell subsets from patients with sepsis on the first and seventh days following admission, as well as 111 healthy controls. The patients were divided into survivors and non-survivors, based on 28-day prognosis.

**Results:**

The analysis showed abnormal distribution and selective depletion of B cells and its subsets in the early stages of sepsis. On day 1, compared with survivors, non-survivors showed significant decreases in the proportion and absolute count of transitional (Tr) B cells, reductions in the proportion of CD5^+^ B cells, and increases in the proportion of double-negative (DN) B cells. On day 7, the proportions and absolute counts of Tr and CD5^+^ B cells significantly decreased whereas the proportion of DN B cells significantly increased in non-survivors. Ninety-four survivors and 15 non-survivors were included in our paired-sample rank-sum test. Compared to day 1, only the survivors showed significant increases in absolute B, Tr B, and CD5^+^ B cell counts by day 7. Multivariate Cox regression analysis showed that the proportion of DN B cells on day 1 (hazard ratio = 1.092 [95% confidence interval: 1.035–1.152], P = 0.001) was a risk factor for mortality, and Kaplan–Meier survival curve analysis showed that patients with proportions of DN B cells > 11.81% on day 1 had poorer prognoses. Receiver operating characteristic curve analysis showed that B cell subset parameters could predict mortality (area under the receiver operating characteristic curve [AUC], 0.741) and enhanced the prognostic value of the Acute Physiology and Chronic Health Evaluation II score (AUC, 0.840).

**Conclusion:**

Our study revealed that deficiencies of B, Tr B, and CD5^+^ B cells, as well as a persistent increase in the proportion of DN B cells, were associated with poor prognosis—and that B cell subsets showed predictive value to mortality. These results provide new insights into the roles of B cell subsets in sepsis, as well as ways to better manage its progression and predict its course.

## 1 Introduction

Sepsis is a dysregulated response to infection that has a high mortality rate worldwide (1). Increasing evidence suggests that its two phases—hyperinflammation and immunosuppression—are dynamic and can occur sequentially or simultaneously. Immunosuppression is considered an important factor affecting mortality during the later stages of the condition (2). Lymphopenia is an important cause of immunosuppression (2, 3). Despite being the basis of immune homeostasis, the role of B lymphocytes in sepsis remains poorly understood. This contrasts significantly with the extensive amount of similar studies investigating the role of T lymphocytes (4).

B cells can be categorized according to their surface markers as transitional (Tr), naïve, unswitched memory (UM), switched memory (SM), plasmablasts, double-negative (DN), and CD5^+^ B cells. Tr B cells occur when immature B cells in the bone marrow begin to migrate to the peripheral blood, which then functionally mature into naïve B cells (5). Naïve B cells differentiate into B-1 and B-2 cells (5). B-1 cells (consisting of B-1a and B-1b) are involved in the innate immune response. These cells secrete naturally low-affinity antibodies without entering the germinal center (GC), and help to establish the first line of defense against infections (6). CD5^+^ B refers to a population of cells that are mainly composed of B-1a (7). B-2 cells are classical antibody-producing cells that are involved in adaptive immunity (8). UM B cells do not undergo class-switching, and produce low-affinity IgM antibodies (9) that retain adaptive potential and can access GC when needed. Thus, this population represents the polyreactive pool of memory B cells (10). In the GC, B cells undergo somatic hypermutation, affinity maturation, and class-switching that produces high-affinity plasmablasts (plasma cells) and SM B cells (10). DN B cells are unique memory B cells that are rarely found in healthy populations, accounting for approximately 5% of total peripheral B cells (11). The exact origins of DN B cells remain unclear, and they may represent a heterogeneous population with distinct functions (12).

B cells not only secrete immunoglobulins, but are also important regulators of the innate and adaptive immune systems, activating T cells through antigen presentation and cytokine production to regulate other immune cells (4, 13). Redistribution, cell depletion, and dysfunction greatly limit B cells’ involvement in the immune response to sepsis (13, 14). Significant reductions in B lymphocyte cells counts have been linked to poor prognoses in sepsis (15, 16), and B cell subsets abnormalities have been reported to be associated with poor outcomes. Dong et al. found that the numbers of immature/transitional B cells and resting memory B cells were significantly decreased in non-survivors of sepsis, and that their combination offers good predictive value in terms of mortality risk in this context (15). Similarly, Duan et al. found that a decrease in the proportions of antibody-secreting cells and memory B cells was associated with higher sepsis-related mortality (17). However, the changes that take place in B cell subsets during sepsis remain unclear. Inconsistent conclusions exist regarding whether the source of B cells is impaired in sepsis (15, 17, 18), and the sepsis-induced changes to the B cell subsets involved in innate immune responses remain to be elucidated in humans (19). In addition, DN B cells are rarely reported in sepsis. Therefore, we analyzed the proportions and absolute counts of seven peripheral B cell subsets and their dynamics in sepsis, and evaluated their associations with patient prognoses.

## 2 Materials and methods

### 2.1 Participants

This prospective disease-control study was conducted between March 2021 and December 2023 at Dongyang People’s Hospital, a tertiary comprehensive university hospital in China. Patients from the emergency ward or intensive care unit were selected, using the following inclusion criteria: (1) age > 18 years; and (2) diagnosis of sepsis or septic shock within 24 h of admission that met the Third International Consensus Definitions. The exclusion criteria were: (1) use of antibiotics for > 2 days; (2) pregnant or breastfeeding women; and (3) patients with autoimmune diseases, human immunodeficiency virus or chronic hepatitis viral infection, hematologic disease, malignancy, or who received immunosuppressive therapy. Age- and sex-matched healthy controls (HCs) who underwent health check-ups were included, using identical exclusion criteria. This study was approved by the Ethics Committee of Dongyang People’s Hospital (approval no.: 2021-YX-143), and all of the participants or their guardians provided written informed consent. A flowchart of the participant selection process for the study is shown in **Supplementary Figure 1**.

### 2.2 Sample collection and flow cytometry

Blood was collected from the patients within 24 h of their admission (day 1), and again at the follow-up (between 5:00 am and 11:00 am on day 7). HCs were sampled once during physical examination. Three milliliters of ethylenediaminetetraacetic acid-anticoagulated fresh peripheral blood were collected. The total number of lymphocytes was determined using a hematology analyzer (XN-9000; Sysmex, Japan) within 3 h, and samples were analyzed using 10-color flow cytometry within 24 h to detect immune cell surface antigens. The monoclonal fluorescent antibodies used are listed in **Supplementary Table 1**. Furthermore, 100 µL of whole blood and premixed antibodies were added to each flow tube. B cell subsets were detected using Navios (Navios; Beckman-Coulter, USA), and data were analyzed using Kaluza software version 2.0 (Beckman-Coulter). The absolute count for each subset was calculated based on the absolute number of lymphocytes and the proportion of the subset. The detected B cells were either CD19 or CD20 positive, and categorized into Tr B cells (CD24^high^ CD38^high^) and plasmablasts (CD24¯ CD38^high^). Based on the differential expression of CD27 and IgD in non-Tr B cells/non-plasmablasts, naïve B cells were defined as IgD^+^CD27^−^, DN B cells as IgD^−^CD27^−^, UM B cells as IgD^+^CD27^+^, and SM B cells as IgD^−^CD27^+^. CD5^+^ B cells were defined as CD19^+^CD5^+^. The gating strategies used are described in **Supplementary Figure 2**.

### 2.3 Data collection

The patients’ day 1 demographic characteristics, primary diagnoses, comorbidities, blood lactate levels, procalcitonin (PCT) levels, C-reactive protein (CRP) levels, Acute Physiology and Chronic Health Evaluation II (APACHE II) scores, and Sequential Organ Failure Assessment (SOFA) scores were recorded using a clinical information system. Microbiological data were obtained from the clinical laboratory, and 28-day all-cause mortality data were obtained using a clinical information system or through follow-up telephone calls.

### 2.4 Statistical analysis

Normally-distributed continuous variables are presented as means ± standard deviations and were compared between groups using the independent-samples Student’s t-test. Non-normally distributed continuous variables are presented as medians (interquartile ranges) and were compared using the Mann–Whitney U test. Categorical variables are presented as numbers (percentages), and comparisons between groups were made using Chi-squared or Fisher’s exact tests, as appropriate. Spearman’s correlation analysis was performed on B cell subsets and clinical parameters. The dynamics of B cell subsets associated with prognosis were analyzed using the paired-samples rank-sum test. Variables with P values of < 0.05 in the univariate test, as well as sex, were included in a subsequent multivariate Cox regression analysis to determine the risk factors for all-cause mortality at 28 days. Multicollinearity among the variables was determined based on the variance inflation factor (VIF). A VIF value of < 10 indicated no multicollinearity between the variables. Optimal cutoff values were calculated using the Jordon index method, and Kaplan–Meier curves were analyzed using the log-rank test. Receiver operating characteristic curves were obtained using APACHE II scores, SOFA scores, B cell subset parameters that differed significantly between survivors and non-survivors on day 1, B cell subset parameters with APACHE II scores as the independent variable, and 28-day mortality as the dependent variables. All statistical analyses were performed using IBM SPSS Statistics version 27.0 and GraphPad Prism 8.0 (GraphPad Software, USA). Statistical significance was set at P < 0.05.

## 3 Results

### 3.1 Clinical characteristics

This study included 154 patients with sepsis and 111 HCs. Sex and age did not differ significantly between the groups. The 28-day mortality rate for the patients was 21.43% (n = 33), with 9.09% (n = 14) dying within 7 days. The non-survivors were older than survivors, exhibited a higher proportion of septic shock, and had higher CRP levels, APACHE II scores, and SOFA scores. The baseline characteristics of the included patients are presented in **Table 1**.

**Table 1:** Baseline characteristics of sepsis population.

Parameter	Total (n = 154)	Survivors (n = 121)	Non-survivors (n = 33)	P value
Age, years	76.00 (66.00–84.00)	75.00 (64.50–83.00)	83.00 (74.50–86.50)	**0.003**
Males (n, %)	97 (62.99%)	76 (62.81%)	21 (63.64%)	0.931
Laboratory test				
WBC (10^9^/L)	10.60 (7.14–16.05)	10.89 (7.08–15.80)	9.60 (7.28–17.36)	0.988
Lymphocyte (10^9^/L)	0.56 (0.31–0.81)	0.59 (0.34–0.81)	0.47 (0.24–0.78)	0.278
Lactate (mmol/L)	2.30 (1.65–3.65)	2.30 (1.60–3.43)	2.70 (1.70–4.20)	0.11
PCT (ng/mL)	7.11 (1.00–23.72)	6.15 (0.90–22.06)	8.52 (3.82–46.39)	0.065
CRP (mg/L)	132.49 (52.09–183.88)	118.13 (44.74–179.87)	160.10 (100.22–207.00)	**0.007**
APACHE II score	13.0 (10.0–19.00)	12.00 (9.00–16.00)	22.00 (15.00–27.50)	**<0.001**
SOFA	7.00 (3.75–11.00)	6.00 (3.00–9.50)	12.00 (8.00–15.00)	**<0.001**
Diagnostic (n, %)				
Sepsis	74 (48.05%)	68 (56.20%)	6 (18.18%)	**<0.001**
Septic shock	80 (51.95%)	53 (43.80%)	27 (81.82%)	
Comorbidities (n, %)				
Cardiovascular disease	44 (28.57%)	34 (28.10%)	10 (30.30%)	0.491
Diabetes mellitus	35 (22.72%)	30 (24.79%)	5 (15.15%)	
Hypertension	86 (55.84%)	73 (60.33%)	13 (39.39%)	
Pathogens (n, %)				
Bacteria	76 (49.35%)	65 (53.72%)	11 (33.33%)	0.07
Fungi	1 (0.65%)	0 (0)	1 (3.03%)	
Bacteria and fungi	6 (3.90%)	4 (3.30%)	2 (6.06%)	
Unknown	71 (46.10%)	52 (42.98%)	19 (57.58%)	

WBC, white blood cell; PCT, procalcitonin; CRP, C-reactive protein; APACHE, Acute Physiology and Chronic Health Evaluation; SOFA, Sequential Organ Failure Assessment. The bold P values indicates statistically significant (P < 0.05).

### 3.2 Redistribution and depletion of B cell subsets in patients with sepsis on admission

The proportions of Tr B cells, UM B cells, SM B cells, plasmablasts, and CD5^+^ B cells were significantly decreased, and the proportions of B cells, naïve B cells, and DN B cells were significantly increased in the patients on admission compared to the HCs (**Supplementary Table 2**). Regarding absolute counts, B cells and other subsets were significantly decreased, except for DN B cells—though not to a statistically significant degree (4.63 [2.65–7.62] vs. 5.87 [3.20–9.73], P = 0.053). The increased proportions of B cells, naïve B cells, and DN B cells in the patients with sepsis were not attributable to increases in their absolute counts, but may have resulted from lower counts for other subsets.

### 3.3 Correlation between B cell subsets and clinical parameters

Clinical severity scores (i.e., APACHE II and SOFA scores) and clinical parameters such as CRP, PCT, and blood lactate levels were measured to reflect sepsis severity. We analyzed data from 154 patients with sepsis on day 1, and our correlation analysis (**Supplementary Table 3**) showed that B cell proportions were positively correlated with SOFA (r = 0.165, P = 0.041) and PCT (r = 0.348, P < 0.001). The proportion of Tr B cells was negatively correlated with APACHE II (r = –0.331, P < 0.001). The proportion of naïve B cells was positively correlated with PCT (r = 0.203, P = 0.021). The proportion of SM B cells was negatively correlated with PCT (r = –0.261, P = 0.003). The proportion of DN B cells was positively correlated with APACHE II (r = 0.185, P = 0.022) and CRP levels (r = 0.202, P = 0.015). The proportion of CD5^+^ B cells was negatively correlated with APACHE II (r = –0.266, P < 0.001) and CRP levels (r = –0.276, P < 0.001). Absolute Tr B cell count was negatively correlated with APACHE II (r = –0.286, p < 0.001). Absolute CD5^+^ B cell count was negatively correlated with APACHE II (r = −0.211, P = 0.009) and CRP levels (r = –0.174, P = 0.037).

### 3.4 B cell subsets associated with survival in patients with sepsis

Compared to survivors, non-survivors of sepsis had significantly lower proportions and absolute counts of Tr B cells, significantly lower proportions of CD5^+^ B cells, and significantly higher proportions of DN B cells on day 1 (**Table 2**), with the difference in absolute CD5^+^ B cells count almost reaching statistical significance (2.10 [0.84–13.84] vs. 5.06 [2.48–13.71], P = 0.071). After excluding patients who withdrew from the study because of transfer, death, or other personal reasons, a total of 109 patients underwent repeat testing on day 7—including 94 in the 28-day survivor group and 15 in the non-survivor group. On day 7, the proportions and absolute Tr B cells and CD5^+^ B cells counts were significantly lower in the non-survivors. The proportions of SM B cells and DN B cells were significantly higher, and the absolute count of naïve B cells showed a decreasing trend that approached statistical significance (33.01 [5.23–88.91] vs. 55.10 [25.93–94.92], P = 0.086; **Table 2**). Moreover, we found that the increased proportions of DN B and SM B cells in the non-survivors were not caused by an increase in their absolute counts.

**Table 2:** Analysis of parameters of B cell subsets based upon mortality.

Parameter	Mortality from day 1			Mortality from day 7		
	Survivors (n = 121)	Non-survivors (n = 33)	P value	Survivors (n = 94)	Non-survivors (n = 15)	P value
B cells (%)	13.68 (8.16–20.99)	16.17 (8.84–26.80)	0.354	10.09 (7.75–15.15)	10.71 (6.52–25.12)	0.788
Transitional B (%)	4.02 (2.25–6.85)	1.38 (0.52–5.57)	**0.001**	3.56 (1.71–6.14 )	0.73 (0.41–2.54)	**<0.001**
Naïve B (%)	71.96 (57.10–79.26)	71.33 (42.55–81.80)	0.563	53.86 ± 17.72	43.47 ± 24.12	0.128
Unswitched memory B (%)	3.85 (2.58–7.70)	4.45 (2.14–7.22)	0.939	6.01 (4.40–8.63)	4.79 (2.74–12.15)	0.654
Switched memory B (%)	8.28 (4.92–16.22)	6.59 (2.67–20.61)	0.289	14.64 (9.72–20.38)	26.56 (16.28–44.95)	**0.006**
Plasmablasts (%)	0.87 (0.35–2.51)	1.92 (0.34–4.91)	0.303	7.65 (3.10–15.10)	8.25 (5.47–19.16)	0.465
DN B (%)	6.79 (4.11–11.25)	10.23 (4.67–15.58)	**0.049**	5.70 (3.71–8.95)	7.78 (4.86–13.3)	**0.04**
CD5^+^ B (%)	8.27 (5.29–13.24)	4.67 (3.30–10.79)	**0.01**	8.72 (5.50–13.49)	4.32 (1.24–6.72)	**<0.001**
B cells (10^6^/L)	68.25 (37.69–124.24)	80.34 (27.87–164.09)	0.942	108.20 (60.96–163.89)	71.35 (27.27–141.76)	0.133
Transitional B (10^6^/L)	2.76 (1.15–6.77)	0.55 (0.21–5.49)	**0.004**	3.59 (1.46–6.64)	0.65 (0.48–1.08)	**<0.001**
Naïve B (10^6^/L)	43.45 (23.17-–92.04)	51.26 (11.82–140.16)	0.814	55.10 (25.93–94.92)	33.01 (5.23–88.91)	0.086
Unswitched memory B (10^6^/L)	2.78 (1.33–6.11)	2.99 (1.22–7.40)	0.939	6.69 (3.10–11.48)	5.53 (0.75–11.44)	0.471
Switched memory B (10^6^/L)	5.95 (3.37–10.67)	4.59 (2.04–10.88)	0.222	14.58 (9.32–22.13)	17.53 (4.99–31.12)	0.61
Plasmablasts (10^6^/L)	0.53 (0.26–1.50)	0.82 (0.35–1.84)	0.25	7.60 (3.04–14.58)	6.91 (2.55–13.67)	0.616
DN B (10^6^/L)	4.65 (2.61–6.91)	4.49 (2.96–12.33)	0.463	6.07 (3.70–10.34)	6.29 (3.63–7.26)	0.93
CD5 ^+^ B (10^6^/L)	5.06 (2.48–13.71)	2.10 (0.84–13.84)	0.071	9.31 (4.27–18.41)	2.17 (0.75–6.60)	**<0.001**

DN, double-negative. The bold P values indicates statistically significant (P < 0.05).

We matched the two groups on the first day in terms of age and sex, with 40 survivors and 28 non-survivors, and found that the non-survivors consistently had lower proportions and absolute counts of Tr B cells, significantly lower proportions of CD5^+^ B cells, and significantly higher proportions of DN B cells (**Supplementary Table 4**).

### 3.5 Dynamics of B cell subsets associated with prognosis

A paired test on days 1 and 7 revealed that the increased proportion of naïve B cells in the patients with sepsis decreased significantly after treatment, both in survivors and non-survivors. The proportions of SM B cells and plasmablasts increased significantly in both groups (**Table 3**). Moreover, the heterogeneous distribution of B cells improved only in the survivors, where the proportion of B cells decreased while that of UM B cells increased significantly. Notably, we observed a slight decrease in the Tr B cell ratio in the survivors, which was attributed to a more pronounced increase in other B cell subsets. In terms of absolute counts, all B cell subsets excluding naïve B cells significantly increased among the survivors. In the non-survivors, only UM B cells, SM B cells, and plasmablasts showed significantly increased absolute counts.

**Table 3:** Dynamic changes in B lymphocyte subsets in survivors and non-survivors on days 1 and 7.

Parameter	Survivors (Day 1) (n = 94)	Survivors (Day 7) (n = 94)	P value	Non-survivors (Day 1) (n = 15)	Non-survivors (Day 7) (n = 15)	P value
B cells (%)	12.30 (7.13–19.27)	10.09 (7.75–15.15)	**0.001**	10.3 (7.04–30.82)	10.71 (6.52–25.12)	0.65
Transitional B (%)	3.72 (2.25–6.45)	3.56 (1.71–6.14)	**0.003**	1.03 (0.49–3.24)	0.73 (0.41–2.54)	0.109
Naïve B (%)	71.68 (58.37–79.77)	55.10 (41.91–67.27)	**<0.001**	74.04 (36.79–82.2)	38.04 (20.61–64.35)	**0.001**
Unswitched memory B (%)	3.78 (2.43–8.17)	6.01 (4.40–8.63)	**<0.001**	4.45 (2.10–8.82)	4.79 (2.74–12.15)	0.191
Switched memory B (%)	9.42 (5.18–16.49)	14.64 (9.72–20.38)	**<0.001**	8.19 (3.72–30.29)	26.56 (16.28–44.95)	**0.001**
Plasmablasts (%)	1.03 (0.33–2.61)	7.65 (3.10–15.10)	**<0.001**	2.96 (0.32–4.62)	8.25 (5.47–19.16)	**0.002**
DN B (%)	6.86 (4.16–10.42)	5.70 (3.71–8.95)	0.058	9.03 (5.79–21.99)	7.78 (4.86–13.3)	0.349
CD5^+^ B (%)	7.98 (5.71–12.70)	8.72 (5.50–13.49)	0.526	5.18 ± 3.45	4.52 ± 2.9	0.25
B cells (10^6^/L)	68.02 (38.34–121.03)	108.20 (60.96–163.89)	**<0.001**	40.1 (16.78–154.37)	71.35 (27.27–141.76)	0.156
Transitional B (10^6^/L)	2.44 (1.17–5.18)	3.59 (1.46–6.64)	**0.007**	0.36 (0.21–1.52)	0.65 (0.48–1.08)	0.609
Naïve B (10^6^/L)	43.35 (22.20–85.13)	55.10 (25.93–94.92)	0.061	16.06 (8.92–121.38)	33.01 (5.23–88.91)	0.733
Unswitched memory B (10^6^/L)	2.44 (1.20–6.11)	6.69 (3.10–11.48)	**<0.001**	1.76 (0.75–4.42)	5.53 (0.75–11.44)	**0.02**
Switched memory B (10^6^/L)	5.76 (3.43–10.94)	14.58 (9.32–22.13)	**<0.001**	4.59 (2.39–12.21)	17.53 (4.99–31.12)	**0.009**
Plasmablasts (10^6^/L)	0.52 (0.26–1.50)	7.60 (3.04–14.58)	**<0.001**	0.75 (0.32–1.85)	6.91 (2.55–13.67)	**0.005**
DN B (10^6^/L)	4.63 (2.58–6.77)	6.07 (3.70–10.34)	**<0.001**	3.94 (2.42–11.69)	6.29 (3.63–7.26)	0.307
CD5^+^ B (10^6^/L)	5.05 (2.51–11.30)	9.31 (4.27–18.41)	**<0.001**	1.87 (0.60–4.95)	2.17 (0.75–6.60)	0.82

DN, double-negative. The bold P values indicates statistically significant (P < 0.05).

### 3.6 B cell subsets and prediction of mortality

**Figure 1:**
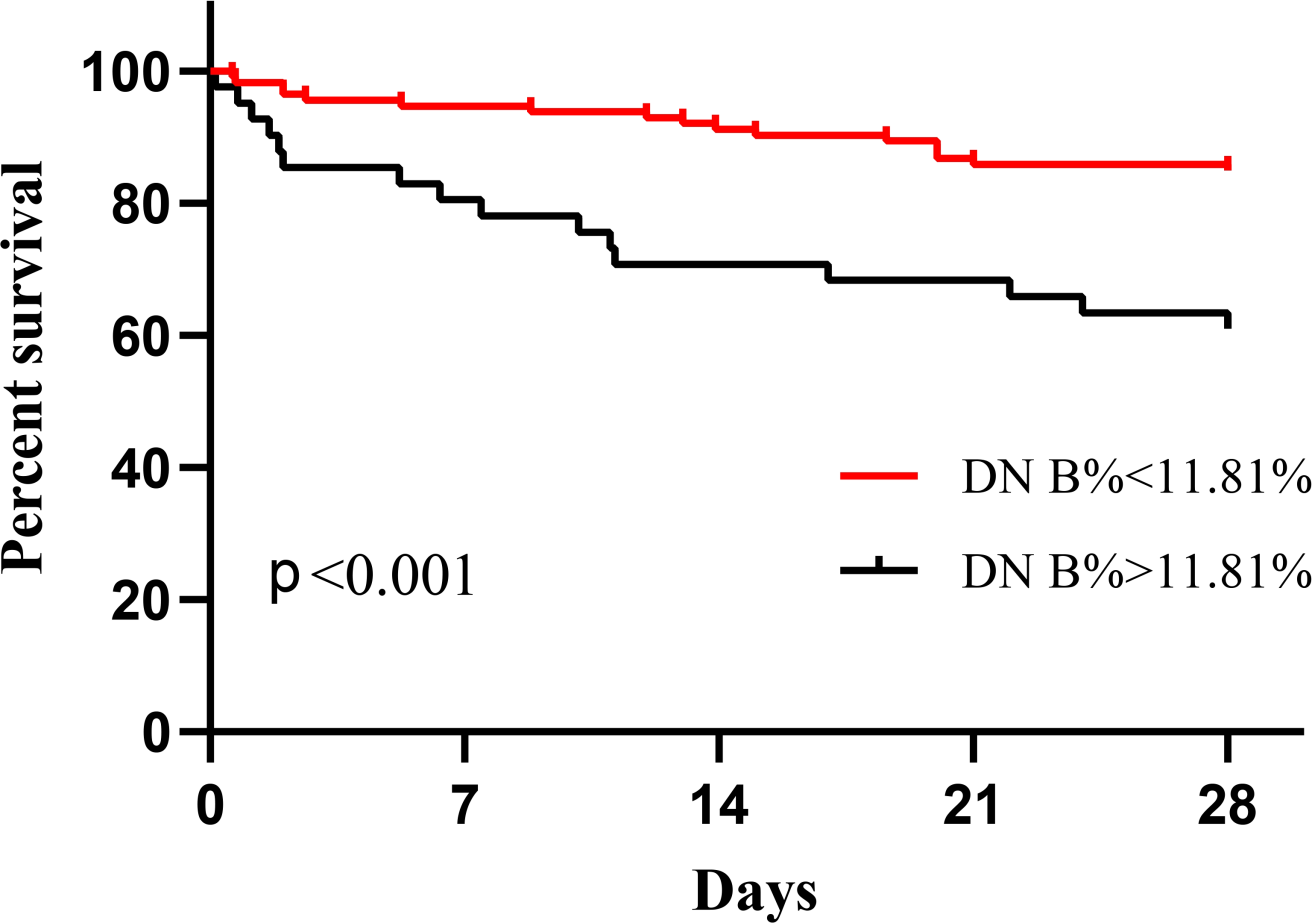
Kaplan–Meier survival curves of proportions of double-negative (DN) B cells at admission.

**Figure 2:**
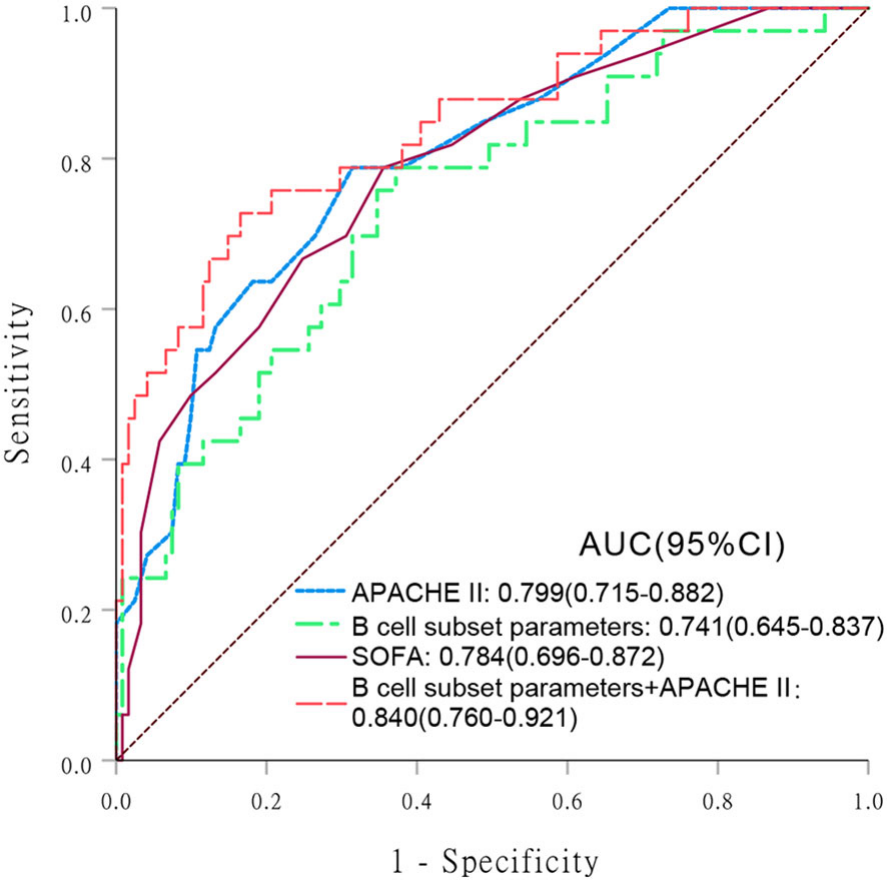
Receiver operating characteristic (ROC) curve analysis comparing the values of various indicators in predicting 28-day mortality risk in patients with sepsis. Areas under the ROC curve (AUC) are presented with 95% confidence intervals (CIs). (1) Acute Physiology and Chronic Health Evaluation (APACHE) II score; (2) B cell subset parameters; (3) Sequential Organ Failure Assessment (SOFA); (4) B cell subset parameters + APACHE II.

The covariates used to test whether B cell subsets were risk factors for 28-day mortality included sex, age, APACHE II score, SOFA score, CRP level, progression to septic shock, proportion of Tr B cells, proportion of DN B cells, proportion of CD5^+^ B cells, and Tr B cell absolute counts. No multicollinearity was identified between the variables according to the results of VIF. Multivariate Cox regression analysis showed that the proportion of DN B cells (hazard ratio = 1.092 [95% confidence interval: 1.035–1.152], P = 0.001) was an independent risk factor for 28-day mortality in patients with sepsis (**Table 4**). Survival curve analysis showed that patients with proportions of DN B cells > 11.81% on day 1 had worse prognoses (p < 0.001; **Figure 1**). The combination of B cell subsets on day 1 (Tr B %, DN B%, CD5^+^ B%, and absolute count of Tr B cells) predicted the risk of death at 28 days (area under the receiver operating characteristic curve, 0.741; 95% confidence interval, 0.645–0.837) and further enhanced the prognostic value of the APACHE II score (area under the receiver operating characteristic curve, 0.840; 95% confidence interval, 0.760–0.921) (**Figure 2**, **Supplementary Table 5**).

**Table 4:** Risk factors for 28‐day mortality in patients with sepsis by multivariate Cox regression analysis.

Variables	Hazard ratio	95% Confidence interval		P value
		Lower	Upper	
APACHE II	1.146	1.091	1.204	< 0.001
Sepsis/septic shock	2.709	0.999	7.349	0.05
DN B (%)	1.092	1.035	1.152	0.001

APACHE, Acute Physiology and Chronic Health Evaluation; DN, double-negative.

## 4 Discussion

Early recognition of immune dysfunction and dynamic monitoring of its changes are important for the effective identification of disease progression, as well as improving prognoses, in patients with sepsis. In this study, through a comprehensive observation of B cells and their seven subsets, we found that their selective depletion, abnormal distribution, and dynamic changes were associated with poor sepsis prognoses. A number of studies have shown that persistent depletion of circulating B cells is associated with poor sepsis prognosis (15, 16), which aligns with our findings. Although the number of B cells decreased, their proportion increased, which may be attributed to a greater decline in T cell numbers (19).

Tr B cells and naïve B cells can further differentiate and develop into other B cell subsets with important and varied roles. We found a significant decrease in absolute counts of Tr B cells and naïve B cells in patients with sepsis compared to HCs, and that a persistent reduction of Tr B cell numbers correlated with poor sepsis prognoses. This suggests that the numbers of Tr B and naïve B cells—the sources of peripheral blood B cells—are reduced during sepsis. We also observed that UM B cells, SM B cells, and plasmablasts decreased significantly during the early stage of sepsis; however, their numbers did not differ significantly between survivors and non-survivors. Moreover, in both groups, the number of UM and SM B cells increased, while that of plasmablasts expanded dramatically on the seventh day—suggesting that their numbers were not a major factor affecting prognosis. Two studies have reported a survival benefit associated with Tr B cells (15, 20), and that decreased naïve B cell numbers were associated with prolonged sepsis (21). Two studies attributed the depletion of B cells to a decrease in antibody-secreting cells and memory B cells, rather than damage to their source (17, 18). These different results may be attributable to the high heterogeneity of sepsis. Variations in pathogens, host-related factors, and degrees of hyperinflammation or immunosuppression contribute to this heterogeneity (22).

The beneficial role of CD5^+^ B cells in sepsis has been demonstrated in several animal experiments. In addition to secreting natural antibodies, these cells also release cytokines that enhance innate and adaptive immune responses, as well as reduce inflammatory reactions—thus suggesting their additional protective roles in sepsis (23, 24). Our study found that the recovery of CD5^+^ B cell populations favors survival in patients with sepsis, and that the proportion and absolute number of these cells was negatively associated with APACHE II and CRP levels. This indicates that the B cell subsets involved in the innate immune response also play an important role in humans, which is closely related to the development and prognosis of sepsis.

The role of DN B cells in infectious diseases has attracted considerable interest (12), and increased proportions have been observed in patients with various infectious diseases such as encephalitis or meningitis (25), malaria (26), and tuberculosis (27). Our findings revealed that proportions of DN B cells were positively correlated with APACHE II scores and CRP levels, and that a sustained increase in the proportion of DN B cells represents an independent risk factor associated with sepsis-related mortality. The increased proportion of DN B cells we observed was attributed to a decrease in other B cell subsets, rather than an increase in their absolute count. In terms of absolute counts, a significant increase was only observed in survivors, with no notable difference between survivors and non-survivors. DN B cells can be categorized into heterogeneous subpopulations with different functions (12). For example, inconsistent trends in DN B cell subsets have been observed in patients with coronavirus disease 2019 (28, 29). Therefore, the specific role of DN B cells in sepsis merits further investigation.

This study was subject to several key limitations worth noting. First, this was a single-center study; thus, further prospective, multicenter studies are warranted to corroborate our results. Second, owing to the difficulty we had in collecting sufficient peripheral blood samples, only the proportion and number of immune cells were examined, without any study of B-cell function. Finally, the timing of the collection of first blood samples was not fixed, and the number of peripheral blood B lymphocytes and their subsets can fluctuate significantly according to circadian rhythms. Therefore, standardizing the sampling time may enhance the reliability of our conclusions.

Our study reveals abnormalities in the numbers of B lymphocytes and their subsets in patients with sepsis that, in turn, affect the relative abundances of specific B cell subsets. A persistent decrease in B lymphocyte, Tr B, and CD5^+^ B cells; an inability to recover from this imbalance; and the persistent increase in the proportion of DN B cells; were all found to be associated with a poor prognosis. The early proportion of DN B cells emerged as an independent predictor of mortality, while the combination of B cell subsets demonstrated predictive value for mortality and further enhanced the prognostic value of the APACHE II score. These results provide new insights into the role of B cell subsets in sepsis, and may better inform clinical judgements regarding disease progression and the management of sepsis.

## Data Availability

The original contributions presented in the study are included in the article/**Supplementary Material**. Further inquiries can be directed to the corresponding author.
