# Supplementary material for: Early B lymphocyte subsets in blood predict prognosis in sepsis
Source: Front Immunol. 2024 Sep 18;15:1437864. doi: 10.3389/fimmu.2024.1437864 (PMC11445034; doi:10.3389/fimmu.2024.1437864)
Supplement: Supplementary file 1 [file DataSheet1.docx]

Supplementary Material

# Supplementary Figures

**Supplementary Figure 1.** Screening of patients with sepsis and healthy controls


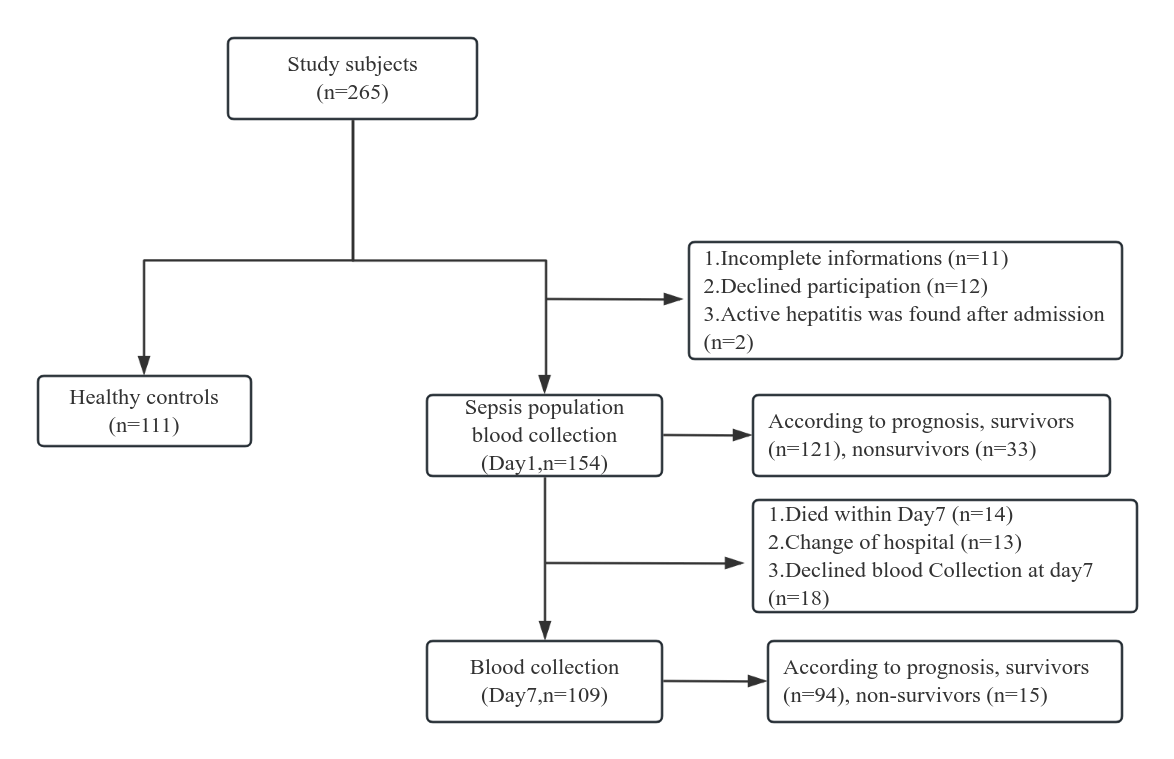


**Supplementary Figure 2.** Gating strategies for identifying circulating immune B cell subsets using flow cytometry. Others = ("B cells" AND (NOT "Transitional B cells") AND (NOT "Plasmablasts").


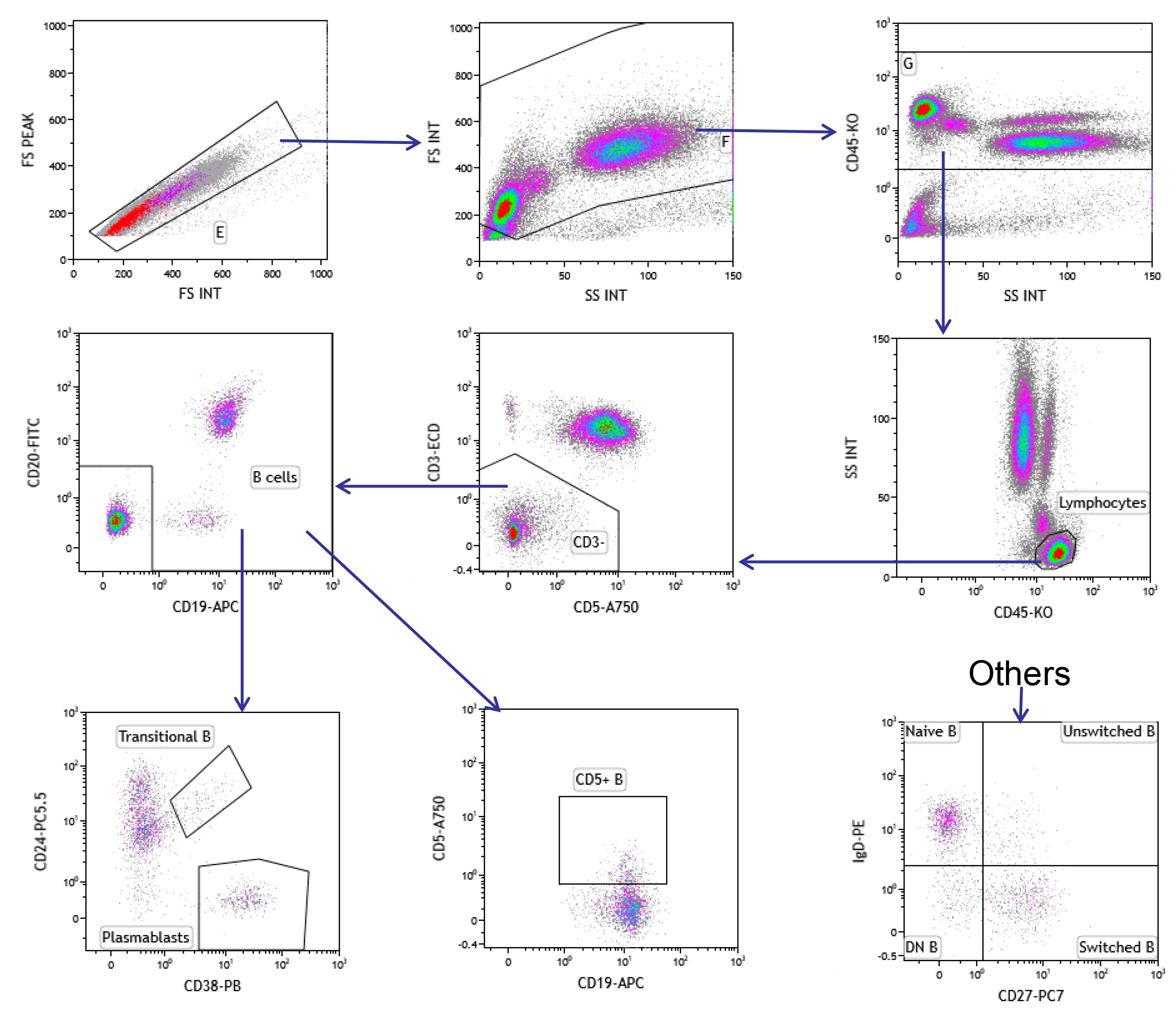


# Supplementary Tables

**Supplementary Table 1.** Monoclonal fluorescent antibody labeling of circulating B cells

| **Marker** | **Fluorochrome** | **Clone** | **Company** |
| --- | --- | --- | --- |
| CD45 | KRO | J.33 | Beckman Coulter |
| CD3 | ECD | UCHT1 | Beckman Coulter |
| CD19 | APC | J4.119 | Beckman Coulter |
| CD20 | FITC | B9E9 | Beckman Coulter |
| CD24 | PE-Cy5.5 | ALB9 | Beckman Coulter |
| CD38 | PB | LS198-4-3 | Beckman Coulter |
| CD27 | PE-Cy7 | 1A4CD27 | Beckman Coulter |
| IgD | PE | IA6-2 | Beckman Coulter |
| CD5 | AA750 | BL1a | Beckman Coulter |

KRO, Krome Orange; ECD, Phycoerythrin-Texas; APC, Allophycocyanin; FITC, Fluorescein Isothiocyanate; PE-Cy5.5, Phycoerythrin-Cyanin 5.5; PB, Pacific Blue; PE-Cy7, Phycoerythrin-Cyanin 7; PE, Phycoerythrin; AA750, APC-Alexa Fluor 750

**Supplementary Table 2.** Analysis of parameters of B cell subsets in healthy controls and patients at admission

| Parameter | Healthy controls  (n =111 ) | Patients at admission  (n = 154 ) | P  value |
| --- | --- | --- | --- |
| Age | 73.00 (66.00–80.00) | 76.00 (66.0–-84.00) | 0.094 |
| Male | 70 (63.06%) | 97 (62.99%) | 0.99 |
| B cells (%) | 8.65 (5.66–12.03) | 13.97 (8.55–21.95) | **<0.001** |
| Transitional B (%) | 7.90 (5.47–11.76) | 3.59 (1.38–6.60) | **<0.001** |
| Naïve B (%) | 59.34 (51.44–64.05) | 71.45 (55.99–79.37) | **<0.001** |
| Unswitched memory B(%) | 9.38 (5.25–13.59) | 3.89 (2.46–7.69) | **<0.001** |
| Switched memory B (%) | 13.84 (11.16–21.55) | 8.17 (4.62–16.49) | **<0.001** |
| Plasmablasts (%) | 1.50 (0.81–2.78) | 0.99 (0.35–3.03) | **0.006** |
| DN B (%) | 4.03 (2.88–6.33) | 7.00 (4.17–12.96) | **<0.001** |
| CD5^+^ B (%) | 15.59 (10.72–21.65) | 7.78 (4.44–12.73) | **<0.001** |
| B cells (10^6^/L) | 149.26 (92.95–220.19) | 68.35 (36.93–131.07) | **<0.001** |
| Transitional B (10^6^/L) | 11.87 (5.91–20.78) | 2.20 (0.64–6.63) | **<0.001** |
| Naïve B (10^6^/L) | 85.08 (48.71–134.79) | 43.94 (20.81–106.06) | **<0.001** |
| Unswitched memory B (10^6^/L) | 11.95 (7.05–20.09) | 2.79 (1.31–6.12) | **<0.001** |
| Switched memory B (10^6^/L) | 19.30 (12.13–31.92) | 5.71 (3.00–10.56) | **<0.001** |
| Plasmablasts (10^6^/L) | 2.30 (1.26–3.42) | 0.58 (0.29–1.61) | **<0.001** |
| DN B (10^6^/L) | 5.87 (3.20–9.73) | 4.63 (2.65–7.62) | 0.053 |
| CD5^+^ B (10^6^/L) | 22.31 (11.66–39.01) | 4.90 (1.86–13.57) | **<0.001** |

DN, double-negative.

The bold P values indicates statistically significant (P < 0.05).

**Supplementary Table 3.**Spearman’s correlation between B cell subsets and clinical parameters on day 1 in patients with sepsis

|  | APACHE II score | SOFA | CRP  (mg/L) | PCT  (ng/mL) | Lactate  (mmol/L) |
| --- | --- | --- | --- | --- | --- |
| B cells (%) | r = 0.086 | **r = 0.165** | r = 0.057 | **r = 0.348** | r = −0.022 |
|  | p = 0.288 | **p = 0.041** | p = 0.495 | **p < 0.001** | p = 0.807 |
| Transitional B (%) | **r = −0.331** | r = −0.09 | r = −0.138 | r = −0.021 | r = −0.078 |
|  | **p < 0.001** | p = 0.269 | p = 0.098 | p = 0.813 | p = 0.378 |
| Naïve B (%) | r = 0.002 | r = −0.05 | r = −0.069 | **r = 0.203** | r = 0.072 |
|  | p = 0.976 | p = 0.541 | p = 0.41 | **p = 0.021** | p = 0.42 |
| Unswitched memory B (%) | r = −0.033 | r = 0.055 | r = −0.054 | r = −0.081 | r = −0.027 |
|  | p = 0.689 | p = 0.5 | p = 0.519 | p = 0.364 | p = 0.76 |
| Switched memory B (%) | r = −0.053 | r = −0.1 | r = −0.053 | **r = −0.261** | r = −0.053 |
|  | p = 0.511 | p = 0.219 | p = 0.523 | **p = 0.003** | p = 0.553 |
| Plasmablasts (%) | r = 0.097 | r = 0.012 | r = 0.119 | r = −0.086 | r = −0.015 |
|  | p = 0.232 | p = 0.884 | p = 0.153 | p = 0.337 | p = 0.87 |
| DN B (%) | **r = 0.185** | r = 0.086 | **r = 0.202** | r = −0.024 | r = 0.025 |
|  | **p = 0.022** | p = 0.291 | **p = 0.015** | p = 0.79 | p = 0.782 |
| CD5^+^ B (%) | **r = −0.266** | r = −0.094 | **r = −0.276** | r = −0.063 | r = −0.072 |
|  | **p < 0.001** | p = 0.247 | **p < 0.001** | p = 0.479 | p = 0.416 |
| B cells (10^6^/L) | r = −0.059 | r = −0.056 | r = −0.011 | r = 0.139 | r = −0.074 |
|  | p = 0.467 | p = 0.49 | p = 0.895 | p = 0.117 | p = 0.402 |
| Transitional B (10^6^/L) | **r = −0.286** | r = −0.111 | r = −0.092 | r = 0.079 | r = −0.095 |
|  | **p < 0.001** | p = 0.172 | p = 0.269 | p = 0.377 | p = 0.286 |
| Naïve B (10^6^/L) | r = −0.044 | r = −0.062 | r = −0.035 | r = 0.157 | r = −0.064 |
|  | p = 0.587 | p = 0.443 | p = 0.676 | p = 0.078 | p = 0.469 |
| Unswitched memory B (10^6^/L) | r = −0.083 | r = −0.008 | r = −0.015 | r = 0.066 | r = −0.092 |
|  | p = 0.304 | p = 0.921 | p = 0.86 | p = 0.457 | p = 0.298 |
| Switched memory B (10^6^/L) | r = −0.114 | r = −0.156 | r = −0.036 | r = −0.05 | r = −0.119 |
|  | p = 0.159 | p = 0.053 | p = 0.671 | p = 0.574 | p = 0.179 |
| Plasmablasts (10^6^/L) | r = 0.058 | r = −0.029 | r = 0.105 | r = 0.006 | r = −0.05 |
|  | p = 0.478 | p = 0.718 | p = 0.208 | p = 0.948 | p = 0.577 |
| DN B (10^6^/L) | r = 0.046 | r = −0.035 | r = 0.136 | r = 0.119 | r = −0.072 |
|  | p = 0.569 | p = 0.664 | p = 0.102 | p = 0.18 | p = 0.415 |
| CD5 ^+^ B (10^6^/L) | **r = −0.211** | r = −0.121 | **r = −0.174** | r = 0.061 | r = −0.076 |
|  | **p = 0.009** | p = 0.134 | **p = 0.037** | p = 0.496 | p = 0.39 |

DN, double-negative; APACHE, Acute Physiology and Chronic Health Evaluation; SOFA, Sequential Organ Failure Assessment; CRP, C-reactive protein; PCT, procalcitonin.

The bold P values indicates statistically significant (P < 0.05).

**Supplementary Table 4.** Analysis of parameters of B cell subsets on day 1 in age-and sex-matched survivors and non-survivors

| Parameter | Survivors  (n =40 ) | Non-survivors  (n = 28 ) | P  value |
| --- | --- | --- | --- |
| Age | 81.00 (71.25–84.00) | 82.00(73.00-84.75) | 0.392 |
| Male | 23(57.50%) | 16 (57.14%) | 0.977 |
| B cells (%) | 15.67(10.64-23.56) | 17.05(9.32-27.89) | 0.99 |
| Transitional B (%) | 4.07(2.54-6.97) | 1.55(0.60-5.61) | **0.006** |
| Naïve B (%) | 72.98(59.87-80.44) | 71.39(42.49-80.87) | 0.376 |
| Unswitched memory B (%) | 3.82(2.70-7.62) | 4.68(2.12 -8.56) | 0.965 |
| Switched memory B (%) | 8.64(4.78-16.40) | 6.02(2.60-20.02) | 0.383 |
| Plasmablasts (%) | 0.86(0.26-1.98) | 1.44(0.33-5.29) | 0.376 |
| DN B (%) | 6.63±3.29 | 10.69±7.70 | **0.013** |
| CD5^+^ B (%) | 10.17(4.89-14.50) | 4.98(3.29-9.42) | **0.01** |
| B cells (10^6^/L) | 84.51(43.87-172.09) | 90.75(35.21-178.32) | 0.794 |
| Transitional B (10^6^/L) | 3.31(1.53-8.02) | 0.98(0.22-8.48) | **0.022** |
| Naïve B (10^6^/L) | 55.92(25.68-127.60) | 55.93(13.27-149.46) | 0.509 |
| Unswitched memory B (10^6^/L) | 3.48(1.34-8.16) | 3.99(1.42-8.61) | 0.975 |
| Switched memory B (10^6^/L) | 7.20(4.22-11.75) | 5.20(2.60-12.07) | 0.202 |
| Plasmablasts (10^6^/L) | 0.62(0.31-1.83) | 0.89(0.35-1.85) | 0.672 |
| DN B (10^6^/L) | 5.01(3.40-6.81) | 4.56(3.05-13.11) | 0.562 |
| CD5^+^ B (10^6^/L) | 6.81(2.71-25.16) | 3.15(0.95-16.21) | 0.058 |

DN, double-negative.

The bold P values indicates statistically significant (P < 0.05).

**Supplementary Table 5.** Receiver operating characteristic curves of the prognostic values of different parameters in sepsis

| Indictor | AUC | 95% CI | Sensitivity | Specificity | P value |
| --- | --- | --- | --- | --- | --- |
| APACHE II | 0.799 | 0.715–0.882 | 0.788 | 0.686 | <0.001 |
| B cell subset parameters | 0.741 | 0.645–0.837 | 0.788 | 0.628 | <0.001 |
| SOFA | 0.784 | 0.696–0.872 | 0.788 | 0.645 | <0.001 |
| B cell subset parameters + APACHE II | 0.840 | 0.760–0.921 | 0.727 | 0.835 | <0.001 |

APACHE, Acute Physiology and Chronic Health Evaluation; SOFA, Sequential Organ Failure Assessment; AUC, Areas under the receiver operating characteristic curve; CI, confidence interval.
